# Aerobic Exercise As a Potential Way to Improve Self-Control after Ego-Depletion in Healthy Female College Students

**DOI:** 10.3389/fpsyg.2016.00501

**Published:** 2016-04-18

**Authors:** Zhiling Zou, Yang Liu, Jing Xie, Xiting Huang

**Affiliations:** Faculty of Psychology, Southwest UniversityChongqing, China

**Keywords:** aerobic exercise, self-control, stamina, pain tolerance, cold pressor task (CPT)

## Abstract

**Purpose:** To test whether aerobic exercise can help build self-control stamina in healthy female young adults. Stamina in this context is defined as the capability to endure ego depletion, which can be measured with a self-control task following another activity also requiring self-control.

**Methods:** Forty-five healthy undergraduate women were randomized to either an experimental group or control group. Participants in the experimental group were required to run in their campus running field for 30 min for a period of 5 weeks. Individuals in the control group were required to do diary entries regarding self-control in their daily lives, also for a period of 5 weeks. Before and after the 5-week intervention, participants completed a pain threshold test, a color word Stroop task and the following Cold Pressor Task (CPT) (with and without a distraction component).

**Results:** There was significant decrease of pain tolerance in session 2 relative to session 1 in the control group, but no such decline was found in the experimental group (though the improvement of pain tolerance was not significant), possibly suggesting successful self-control against this kind of decline.

**Conclusions:** Five weeks of aerobic exercise increased self-control after ego depletion in terms of pain tolerance. These findings suggest that aerobic exercise may serve as a potential effective intervention for enhancing self-control in a college female population.

## Introduction

Self-control involves the ability to filter irrelevant environmental information, the ability to override a pre-potent response, or stop an ongoing response, and plays a vital role in daily life (Barkley, 1997; Davidson et al., 2006). Recently, Berkman et al. (2012) put forward a multi-dimensional perspective of self-control. According to their theory, self-control can be subdivided into three parts: behavioral control (e.g., stopping at a green light for a jaywalking pedestrian), emotional control (e.g., controlling one's anger at a demeaning superior at work), and cognitive control (e.g., focusing one's thoughts on the task at hand instead of daydreaming). Exerting self-control brings people's responses into line with certain standards such as ideals, values, morals, and social expectations, and supports the pursuit of long-term goals. On the contrary, many behavioral problems such as drug addiction, eating disorders, and domestic violence involve a lack of self-control (Baumeister and Heatherton, 1996; Baumeister, 2002).

Another related and important theory is the “strength model” of self-control. According to this model, all self-control activities depend on a limited resource, and when a situation demands two consecutive acts of self-control, performance on the second one is frequently impaired. This is usually referred to as ego depletion (Baumeister, 2002; Baumeister et al., 2007). Based on the strength model, there are two forms of self-control abilities that can be trained: power (the baseline capacity), and stamina (the capability to endure depletion) (Muraven et al., 1999). In daily life, binge eating, crime, violent acts and addictive relapses tend to occur later in the day, a phenomenon possibly attributed to a depletion of self-control resources after completion of daytime activities (Baumeister, 2002).

Given the vital role of self-control, identifying proper ways to promote it can be of great value, especially ways to improve self-control stamina. In the long run, both self-control power and stamina can be trained and enhanced through small but regular exercise. For example, Muraven et al. (1999) found that 2 weeks of regulating mood or monitoring posture could build self-control stamina, as reflected by improved performance on the hand-grip task after depletion from a former thought-suppression task. Muraven (2010) also found that 2 weeks of cutting back on sweets could improve the overall power of self-control ability measured by a stop signal task.

Among various training protocols, aerobic exercise has been suggested as an effective intervention. Aerobic exercise has been found to have robust (but selective) benefits for executive-control processes in aged people (Colcombe and Kramer, 2003), children, and schizophrenia patients and patients suffering from disease (Franco-Martin et al., 2013; Sollerhed et al., 2013; Li et al., 2014; Yau et al., 2014). However, little is known whether long-term aerobic exercise training can modulate cognitive inhibition in healthy young adults, a population particularly at risk for a variety of impulsive behaviors. Oaten and Cheng (2006a) showed that participants who did physical exercise (aerobic exercise, free-weights or resistance training) had significant improvements in cognitive stamina (enhanced performance) as measured by a visual tracking test following a thought-suppression task. Later, Baker et al. (2010) found that 6 months of high intensity aerobic exercise improved women's baseline self-control performance. However, Baker et al. (2010) did not test self-control after depletion. Overall, there is a need for more empirical studies regarding self-control *after* depletion in order to verify the influence of physical exercise on self-control stamina.

Furthermore, previous studies tend to only test the cognitive component of self-control, and not other kinds of self-control despite this faculty being multi-dimensional in nature that also includes emotional control and behavioral control besides cognitive control. Up to now, possible improvements in behavioral and emotional self-control through physical exercise remain unknown. However, some research hints at possible benefits of physical exercise on emotional and behavioral control. For example, some clinical studies have reported that exercise training can help increase pain tolerance ability in patients with chronic pain (Hayden et al., 2005; Hoffman et al., 2005). As we know, pain is not simply a sensation detected by receptors, but is rather a complex phenomenon accompanied by a series of reactions. Pain tolerance is closely related to emotional (fear of pain) and behavioral (automatic avoidance from pain) self-control (Fernandez and Turk, 1992). Thus, pain tolerance may be a good index to test whether physical exercise can modulate emotional and behavioral self-control in a healthy population.

A recent report of the benefits of aerobic exercise on pain tolerance seems promising (Jones et al., 2014). That study found that 6 weeks of structured aerobic exercise training increased ischemic pain tolerance in healthy individuals. Though the Jones study provided evidence that aerobic exercise could lead to increased pain tolerance, several limitations confined the generalization of their findings. Firstly, the sample size was relatively small (12 in the training group and 12 in the control group), and the sample spanned a large age range (18–50 years old). Secondly, there was no intervention for the control group, which could have enlarged the effect of the manipulations because of potential placebo effects. Thirdly, the author only investigated pain tolerance at baseline to test for self-control ability, lacking the stamina component of self-control revealed after depletion.

In the present study, we intended to explore whether aerobic exercise could improve pain tolerance (as an index of behavioral and emotional self-control) after ego-depletion in healthy young adults. In order to measure stamina, we used the color word Stroop task (Stroop, 1935), a well-established and effective method for causing depletion (Wallace and Baumeister, 2002). Pain tolerance was measured with the Cold Pressor Task (CPT), which indexes people's self-control ability to retain their hand immersed in cold water (Kanfer and Seidner, 1973). Shorter immersion duration on this task has been associated with poor self-control (Oosterman et al., 2010). We hypothesized that compared to the control group, the experimental group would have higher pain tolerance in the CPT following ego-depletion.

## Materials and methods

### Participants

Forty-five sophomore female volunteers from Southwest University (Chongqing, China) were recruited from a general psychology course with 50 Yuan RMB for their participation. They were randomly assigned to two groups, with the experimental group containing 22 participants and the control group 23. There were no significant differences between the two groups in age, or years of education (all sophomore students).

The final experimental group only contained 18 participants, and the control group 18, due to 6 participants (two from the experimental group, and four from the control group) giving up during the intervention, and three participants (two from the experimental group, and one participant from the control group) not taking part in post-training tests. Reasons reported for stopping included lack of time/time constraints, academic burden, and absence from school.

Only female students were recruited in the present study due to the following concerns. First, physical exercise frequency, duration, and intensity of Chinese female students has been reported to be significantly lower than those of male students (Zhang et al., 2009). This diversity of physical exercise baseline would make it hard to discern appropriate exercise intensity for each gender during the training. Second, females tend to have shorter pain tolerance during performance of the Cold Pressor Task (CPT), thought to be due to a decrease in pain threshold when reaching puberty (Fillingim et al., 2009; Schmitz et al., 2013; Sollerhed et al., 2013). Thus, only female participants were recruited to attain a more homogeneous sample in order to minimize individual differences. Last but not the least, gender is a factor needed to be considered in the CPT because it may influence the pain tolerance performance in CPT (Fillingim, 2000). For instance, Levine and De Simone (1991) found a significant interaction of experimenter gender and subject gender on pain tolerance; subjects tolerated pain longer when tested by an experimenter of the opposite sex. The experimenters of this study were females, and so only female subjects were recruited in order to avoid an interaction of gender.

### Ethics statement

This study was approved by the review board and Ethics Committee of Southwest University. Written informed consent was obtained from all participants. All participants were informed that their participation was completely voluntary and that they may withdraw from it at any time. All participants were over 18 years of age.

### Materials and tasks

#### Physical activity rating scale (PARS-3)

The PARS-3(Hashimoto, 1990) evaluates one's physical activity by the multiplication of exercise intensity, frequency and time, with the resulting score ranging from 0 to 100. The Chinese version of the PARS-3 has a high reliability and validity in Chinese samples (Liang and Liu, 1994).

#### Color word stroop task

The color word Stroop task, a well-established cognitive test of inhibition, was used to create depletion. Color words presented included RED, GREEN, BLUE, and YELLOW (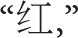 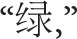 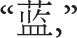 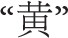 in Chinese, respectively). Print colors in which the words could be displayed were the same as the traditional task. A non-color word, “ke” (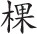), printed with the above four colors, was treated as the neutral word. Such a task conducted with Chinese words maintains the essence of the original task, and is widely used by local Chinese psychologists (Li et al., 2009).

After practice, 216 trials were presented in random order, including 72 congruent trials (e.g., the word RED printed in red), 72 incongruent trials (e.g., the word RED printed in green), and 72 control trials (e.g., the Chinese word “ke” printed in red). In each trial, a central fixation lasting for 300 ms, a black screen for 300 or 500 ms randomly, and stimuli for 1200 ms, were presented in sequence. Participants were required to respond accordingly to the color of the ink, while ignoring the words' semantic meaning (i.e., if the word BLUE appeared in red, they should respond “red”). They were required to respond as quickly as possible by pressing “F,” “G,” “H,” or “J” on the keyboard, for red, yellow, green, and blue, respectively. As the subjects' tendency is to name the word itself (rather than the color of the text), the Stroop task requires cognitive control and an ability to focus one's attention, causing self-depletion (Wallace and Baumeister, 2002).

#### Pain threshold test

We measured the pain threshold of participants with a pain threshold test. Participants were instructed to immerse their non-dominant hand in a circulating bath of cold water (5°C), and were given the following instructions:

*The water temperature is very low, and the sensation may be somewhat unpleasant. However, I would like to emphasize that this procedure is in no way harmful. First, you will immerse your non-dominant hand into the cold water. At the moment you feel pain, please shout out and withdraw your hand out of the water*.

The duration of immersion (in milliseconds, recorded by a stop watch), from the time the hand was placed into the water to the moment of pain perception, was used as the index for baseline pain threshold.

#### Cold pressor task I

We measured the pain tolerance of participants using the Cold Pressor Task (CPT). Participants were instructed to immerse their non-dominant hand in a circulating bath of cold water (5°C), and to hold it there for as long as possible with the following instruction (von Baeyer et al., 2005):

*Now, you will try the cold water one more time. This time, please try your best to keep your hand in the cold water as long as you can or until you are required to withdraw. The longer you keep your hand in the water, the more information we can gather, and the more valuable your participation will be. However, when you cannot tolerate the cold any longer, you should withdraw your hand*.

The Cold Pressor Task (CPT) provides a useful measure of pain tolerance (Kanfer and Seidner, 1973). The duration of immersion (in milliseconds, recorded by a stop watch), from the time the hand was placed in the water to when it was voluntarily withdrawn, was the index of pain tolerance. We set 2 min as the time limit in order to not cause physical discomfort to subjects for an extended period of time. Furthermore, after the participants had withdrawn their hand or reached the 2 min limit, they were asked to rate the pain intensity on a Visual Analog Scale (VAS), measuring 100 mm-long anchoring at one end with “0, no pain at all” and “10, unbearable pain” at the other. Ratings of these kinds are regarded as a fast and reliable way to record self-reported pain sensitivity (Turk et al., 1983).

#### Cold pressor task II

The Cold Pressor Task II was designed to test pain tolerance while participants are less focused on pain, compared to the Cold Pressor Task I. Here, participants were required to do the same thing as in Cold Pressor Task I, but were additionally asked to complete the color word Stroop task at the same time. Thus, they had to modulate their attention from pain tolerance toward the demands of the Stroop task.

### Procedures and interventions

This was a longitudinal study with two sessions. Before training (i.e., Session 1), all participants were required to complete the baseline tests, including the PARS-3, color word Stroop task followed by the pain threshold test, pain Cold Pressor Task I and Cold Pressor Task II. Presentation of the two pain cold pressor tasks was counter-balanced between subjects. Upon completion, both the experimental group and control group were intercepted by a 5 weeks long intervention after which, all participants were required to come back to the lab to complete the post-training test (i.e., Session 2), which was the same as Session 1.

We chose physical running exercise as the intervention for the experimental group to see whether aerobic training could build self-control ability. The control group was required to keep a diary of exerted self-control in their daily lives. This method was adopted as the intervention for the control group from Muraven's study, where keeping such a diary was shown to increase the saliency of self-control, without actually exerting it (Muraven, 2010).

To be more specific, exercise involves planned, structured, and repetitive activity, improvement in cardiopulmonary fitness, and is performed at least 2 to 3 times a week, for at least 15–20 min on each occasion (Turk et al., 1983). Participants in the experimental group spent 20–30 min running together with the experimenter every evening. The physical training lasted 5 weeks. On the other hand, participants in the control group completed the diary of successful self-control every day. Enough details enclosed were required. For example, wanting a sweet but choosing to not have one, or resisting the thought of giving up on memorizing few vocabulary words, were some examples. Every evening, they handed in their diary and got a new sheet of paper for their next entry from the experimenter.

Furthermore, in Session 2, all participants were required to answer a question, “To what extent do you believe the assigned task could help you build self-control strength?,” on a Likert scale (0 “not at all” and 10“extremely yes”). This question was intended to examine whether our manipulation was successful in letting both groups believe that their assigned intervention could help them build self-control abilities to the same extent.

### Data analysis

Pain sensitivity and pain tolerance were treated as the dependent variables. Pain sensitivity was indexed by the pain threshold and pain rating scores in the Visual Analog Scale (VAS). The pain tolerance was indexed by the duration of immersion that participants kept their hand in the cold-water bath. We set 2 min as the longest limit to prevent potential harm to participants. For those people who did not withdraw their hands from the cold water until 2 min, we set 2 min as their immersion duration when doing the analysis. As the data of tolerance time in this study violated key assumptions of normality, Log transformation was conducted before further analysis (von Baeyer et al., 2005).

We first analyzed the performance of the Cold Pressor Task I and Cold Pressor Task II, respectively. A repeated measures ANOVA was used for the pain tolerance in both sessions. After that, we also conducted a two-sample *t*-test of pain tolerance change across sessions (change = Pain tolerance__post−training_– Pain tolerance__pre−training_) to test for group differences. Then, an ANOVA was used to compare the difference between Cold Pressor Task I and Cold Pressor Task II. At last, a Pearson correlation analysis was used to test the correlation between pain tolerance and PARS-3 scores.

## Results

### Manipulation check

The intervention stage lasted for 5 weeks. However, owing to rainy weather, female students' physiological cycle, and holidays, the average number of times the experimental group engaged in aerobic exercise was 12.89 (*SD* = 4.35), whereas the control group handed in an average of 22.6 (*SD* = 4.77) sheets of diaries. Thus, there was a difference between the number of times the two groups engaged in the assigned activities due to some objective factors [*t*_(36)_ = 6.53, *p* < 0.01, *d* = 2.18].

The scores of the PARS-3showed that the two groups did not differ in their pre-training levels of physical activity [*t*_(34)_ = 0.949, *p* = 0.350), while after intervention, participants in the experimental group scored much higher than those in the control group [*t*_(34)_ = 4.94, *p* < 0.001], indicating that the control group did not engage in as many physical activities as the experimental group did during the intervention (see Table 1). Repeated measures ANOVA of PARS-3 verified the significant group × session interaction [*F*_(1, 34)_ = 20.73, *p* < 0.001]. Furthermore, simple effect analysis showed that the PARS-3 score increased significantly [*F*_(1, 34)_ = 21.913, *p* < 0.001, η^2^ = 0.392] in the experimental group but declined significantly in the control group [*F*_(1, 34)_ = 3.090, *p* > 0.05, η^2^ = 0.083].

**Table 1:** **Mean and SD of demographics and PARS-3 score of participants**.

	**Exp. Group**		**Control Group**		***t***	***p***
	***M***	***SD***	***M***	***SD***		
N	18		18			
Age	20.5	1.01	20.6	1.20	−0.22	0.82
PARS-3 (pre)	11.72	8.96	14.78	10.32	−0.95	0.35
PARS-3 (Post)	25.33	11.2	9.67	7.45	4.94	0.000

*PARS-3, Physical Activity Rating Scale*.

Furthermore, no difference on the question “To what extent do you believe the assigned task could help build your self-control strength” was found between the two groups [*t*_(34)_ = 1.56, *p* = 0.13, *d* = 0.51]. This indicated that participants in the control group also believed they could build their self-control abilities through diary keeping, without actually exerting self-control.

### Pain threshold

The pain threshold was measured before cold pressor tasks in both sessions. A repeated measures ANOVA analysis showed no significant main effect or interaction (*F*s < 1), which indicated the two groups' sensation of pain were similar, pre-(Exp. group: 8.24 ± 0.51; Control group: 8.57 ± 0.43) and post intervention(Exp. group: 8.34 ± 0.45; Control group: 8.40 ± 0.64).

### Pain intensity rating

Pain intensity was measured by VAS rating for the cold pressor tasks across sessions. ANOVA analysis showed no significant main effects nor interactions (*F*s < 1), which showed that the two groups had similar subjective pain experiences pre-(Exp. group: 7.11 ± 0.37; Control group: 8.19 ± 0.33) and post intervention (Exp. group: 7.33 ± 0.29; Control group: 6.97 ± 0.25).

### Pain tolerance in cold pressor tasks

**Figure 1:**
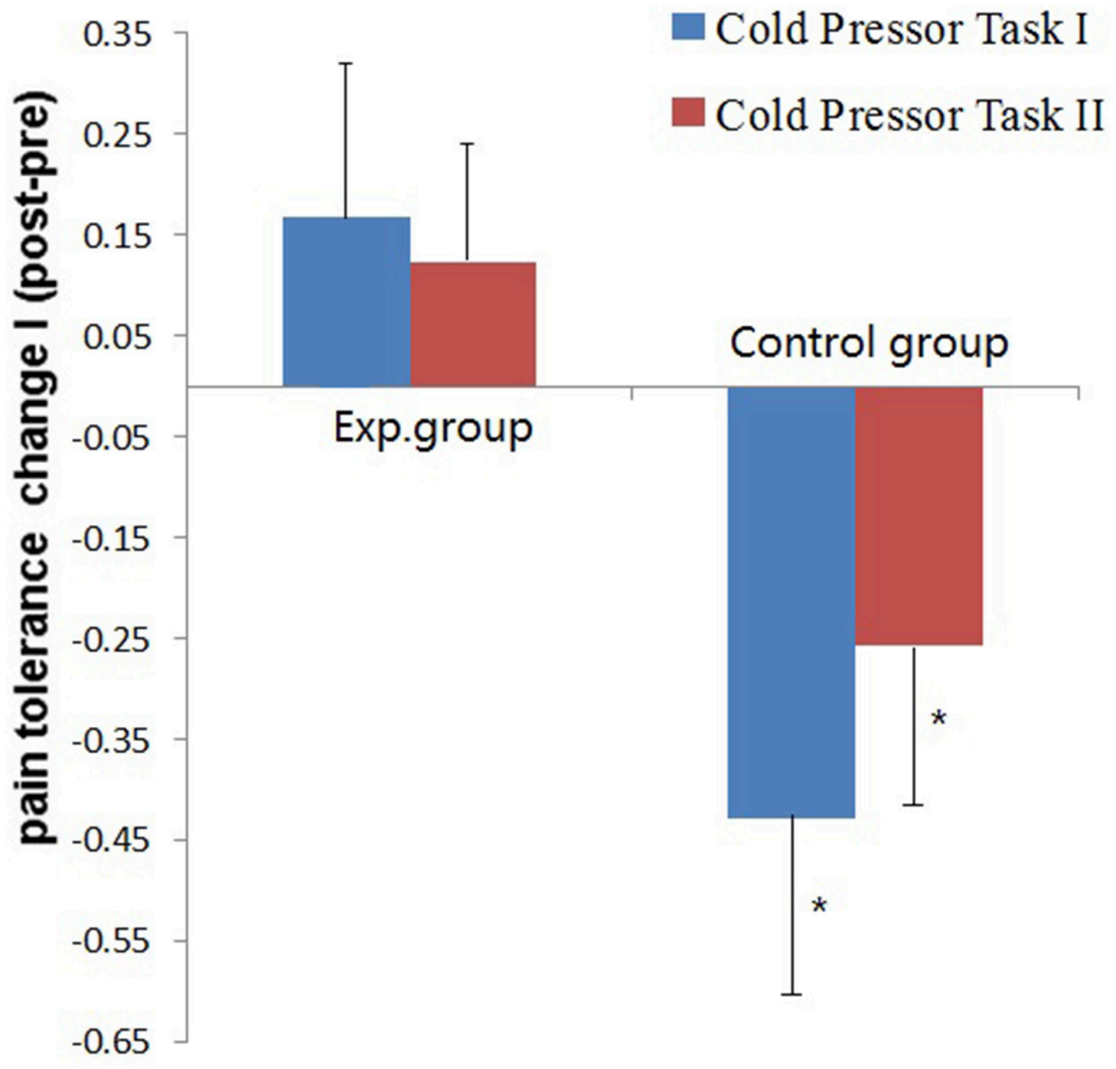
**Mean pain tolerance scores change and S.E.M. of the two groups (Exp. vs. Control group) in the two Cold Pressor Tasks**. Pain tolerance was calculated by the Log_e_ transformation of immersion duration in cold water. Change = Pain tolerance__post−training_ – Pain tolerance__pre−training._^*^p < 0.05.

The mean and SD of pain tolerance in CPTs are shown in Table 2. For Cold Pressor Task I, a repeated measures-ANOVA analysis showed no significant main effect of session (Pre vs. Post) and intervention (Running vs. Daily writing), while the interaction was significant [*F*_(1, 34)_ = 6.57, *p* = 0.015, η^2^ = 0.162]. A further simple effect analysis showed that the control group had a significant decline of pain tolerance across sessions [*F*_(1, 34)_ = 6.80, *p* = 0.013, η^2^ = 0.167]. However, improvement of pain tolerance in the experimental group across sessions was not significant (*F*_(1, 34)_ = 1.03, *p* = 0.317, η^2^ = 0.029]. Furthermore, we did an independent samples *t*-test for the changes of pain tolerance across sessions and verified that the control group had significantly more deterioration [*t*_(34)_ = 2.56, *p* = 0.015], (see Figure 1).

**Table 2:** **Mean and SD of pain tolerance in cold pressor tasks (Log_*e*_ transformed)**.

		**Exp. group**			**Control group**		
		***N***	***M***	***SD***	***N***	***M***	***SD***
CPT I	Pre-training	18	10.01	0.92	18	10.38	1.00
	Post-training	18	10.18	0.94	18	9.95	0.88
	Change(post-pre)	18	0.17	0.15	18	−0.43^*^	0.17
CPT II	Pre-training	18	10.71	0.87	18	10.85	0.79
	Post-training	18	10.83	0.95	18	10.59	0.86
	Change(post-pre)	18	0.12	0.12	18	−0.26^*^	0.13

* *p < 0.05*.

We performed the same analysis to pain tolerance scores in Cold Pressor Task II and got very similar results (see Table 2) though the effect size was much smaller. The session in (Pre vs. Post) × intervention (Running vs. Daily writing) interaction was significant [*F*_(1, 34)_ = 4.25, *p* = 0.047, η^2^ = 0.111]. The group difference of changes of pain tolerance across sessions was also significant [*t*_(34)_ = 2.06, *p* = 0.048], (see Figure 1).

A 2 (task: CPT I vs. CPT II) × 2 (group: Exp. vs. Control) × 2 (session: Pre vs. Post) repeated measures ANOVA analysis was used to find differences between Cold Pressor Task I and Cold Pressor Task II. We found a significant main effect of task [*F*_(1, 36)_ = 68.549, *p* < 0.001, η^2^ = 0.656], showing longer pain tolerance in CPT II in both pre (*p* < 0.001, η^2^ = 0.598) and post training sessions (*p* < 0.001, η^2^ = 0.584). We also found significant session × group interaction [*F*_(1, 36)_ = 7.172, *p* = 0.011, η^2^ = 0.166], similar as shown above.

### Correlations between pars-3 and pain tolerance change

Pearson Correlation analysis showed that the pain tolerance change (Pain tolerance__post_– Pain tolerance__pre_) was significantly positively correlated with the change of PARS-3 scores. In CPT I and CPT II, the correlation coefficient was 0.494 (*p* = 0.002) and 0.333 (*p* = 0.047), respectively.

## Discussion

Previous research studies have supported that aerobic exercise can help with cognitive executive control abilities in elderly, children, and some clinical populations, yet little evidence is given in healthy populations. Furthermore, previous studies focus on the effect of physical exercise on cognitive self-control, with little attention paid to other aspects (emotional and behavioral components) of self-control. Additionally, physical exercise has been shown to improve overall self-control ability, but little is known about its effect on the stamina of self-control. To our knowledge, this is the first study investigating the possible effects of aerobic exercise on the stamina of self-control (pain tolerance as an emotional and behavioral aspect of self-control) in healthy adults.

Our data suggest that after 5 weeks of running, the sensation of pain (Pain threshold and Pain intensity rating) did not change in both groups, but pain tolerance (index of self-control ability) changed significantly. There was significant decrease of pain tolerance in session 2 relative to session 1 in the control group, but no such decline was found in the experimental group (though the improvement of pain tolerance was not significant), possibly suggesting successful self-control against this kind of decline. Furthermore, a significant positive correlation was found between the change of PARS-3 scores and the change of pain tolerance across sessions. These findings suggest that the stamina of self-control may be improved with 5 weeks of aerobic exercise.

### Self-control and pain tolerance

Theoretically, pain can be understood as a subjective, unpleasant experience with both sensory and emotional components [IASP (International Association for the Study of Pain Task Force on Taxonomy), 1994]. Painful stimulation produces general autonomic arousal such as changes in respiration rate, muscle tension, intensification of electro dermal activity, and dilation of the pupils, resulting in behavioral escape from the painful stimuli (Kyle and McNeil, 2014). To combat this “bottom-up” process, the individual must deliberately use central cognitive resources to execute pain control or redirect attention away from pain (Legrain et al., 2009). Self-control is thus vital for this “top-down,” intentional, goal-directed, and effortful process.

Studies have shown that the experience of pain can be influenced by behavioral inhibition (Karsdorp et al., 2014; Pulvers et al., 2014), as in Karsdorp's et al. (2014) study that measured response inhibition with the stop-signal task, along with pain-related fear with the Fear of Pain Questionnaire. Their findings suggest that individuals with stronger response inhibition abilities are better able to inhibit escape/avoidance responses elicited by pain. Verhoeven et al. (2014) found similar results when investigating the role of executive function on pain control in children. Moreover, high self-efficacy regarding the ability to exert control over pain has been shown to result in a significant reduction in anticipated pain intensity, anticipated pain unpleasantness, and experienced pain intensity ratings (Vancleef and Peters, 2011).

Besides behavioral inhibition, studies have also confirmed that the experience of pain can be influenced by emotional factors, such as anxiety (Liang and Liu, 1994), emotional regulation (Tang et al., 2008; Hampton et al., 2015), and emotional intelligence (Ruiz-Aranda et al., 2011). Tang et al. (2008) for example, showed that experimentally induced negative mood increases self-reported pain and decreases tolerance for a pain-relevant task. Ruiz-Aranda et al. (2011) found that participants with higher Emotional Intelligence rated pain as less intense and perceived it as less unpleasant. More recently, Hampton et al. (2015) showed positive effects of emotion regulation strategies for improving pain tolerance, potentially due to the process of reducing the level of negative affect generated by the experimental task. All of this evidence supports that the ability to control the perception of pain requires cognitive strength, and that pain tolerance is closely related to emotional self-control during induced pain.

Moreover, an individual's past experience with pain, the memory of that pain, and the recurrence of pain can lead an individual to anticipate more pain, can impact the amount of fear felt, and can greatly increase pain–avoidance behaviors (Lethem et al., 1983; Turk and Wilson, 2010; Crombez et al., 2012; Vlaeyen and Linton, 2012). According to the fear-avoidance model (or FA model) of pain (Lethem et al., 1983), if an individual experiences acute discomfort and delays the situation by using avoidant behavior, a lack of pain increase reinforces this behavior. Increased vulnerability provides positive feedback to the perceived level of pain, and rewards avoidant behavior for removing unwanted stimuli. If the individual perceives the pain as non-threatening or temporary, he or she feels less anxious and confronts the pain-related situation. In 1993, Waddell et al. developed a Fear-Avoidance Beliefs Questionnaire (FABQ) which showed that fear-avoidance beliefs about physical activities are strongly related to work loss (Waddell et al., 1993).

Thus, the significant decline of pain tolerance in Session 2 for the control group may be attributed to the negative experience of the Cold Pressor Task (CPT) felt in Session 1. However, no such decline was found in the experimental group, possibly suggesting successful self-control against this kind of decline.

### Physical exercise as a way to improve self-control

Overall, the present findings support previous findings that self-control ability can be improved through physical exercise (Taylor et al., 1985). For instance, Smiley-Oyen found that 10 months of aerobic exercise training significantly improved older adults' (65–79 years-old) performance on the Stroop task (Smiley-Oyen et al., 2008), and a similar study found that 6 months of high intensity aerobic exercise improved women's performance on multiple executive function tests, including the Stroop task (Baker et al., 2010). In comparison to previous studies, the present study helped look for potential benefits of physical exercise beyond measures of baseline cognitive self-control.

In terms of the strength model of self-control, self-control is energized by the same metaphorical resource or strength for which the capacity is limited (Baumeister et al., 1998). After a primary act of self-control, this resource can be temporarily depleted (a state termed *ego depletion*). It is important to note however, that ego depletion is not domain specific, meaning that exerting self-control in one domain (e.g., cognitive control in Stroop task) can have an effect on self-control on seemingly unrelated domains (e.g., emotional regulation in Cold Pressor Task) (Baumeister et al., 1998). For example, a series of studies conducted by Megan Oaten showed that after people exercised self-control through financial monitoring or after forcing themselves to study for extended periods of time (e.g., 2 weeks to 4 months), they showed less depletion after completing an unrelated self-regulatory task (Oaten and Cheng, 2006b, 2007). Thus, improvements gained within one domain may be transferable to another (Berkman et al., 2012). The present findings support that improvement gained from aerobic exercise may help enhance self-control in pain tolerance.

Previous studies provide hints to the effect that physical exercise can have on pain tolerance. A school-based study comprised of 206 Swedish children 8–12 years old, showed that physically active children had higher fitness levels and reported less pain symptoms than inactive peers (Sollerhed et al., 2013). Similarly, meta-analyses of studies reveal that athletes possess higher pain tolerance compared to normally active controls (Tesarz et al., 2012). However, only one study directly explores the causality between physical exercise and pain tolerance. Jones et al. (2014), found that pain tolerance in healthy individuals increased after 6 weeks of structured aerobic exercise training. However, our results did not show significant improvement of pain tolerance as Jones et al. (2014) did. We in fact found a significant decline of pain tolerance across sessions in the control group but not in the experimental group.

This inconsistence might be attributed to several factors. The first contributor is the different tasks used in the studies. What Jones et al. (2014) tested was baseline self-control (power), but what we tested was stamina of self-control (i.e., pain tolerance after depletion from the Stroop task). There is no such evidence of the effect of exercise on pain tolerance after ego-depletion. Thus, more empirical studies are needed to confirm our findings. Another factor is that the negative experience of pain may cause shorter pain tolerance in CPT, which is discussed above in terms of the fear-avoidance model of pain(Lethem et al., 1983). The last potential factor that may have contributed to the decline of pain tolerance in the control group was the cold weather. The present study was conducted in winter. The training session began in November and ended in December. With the weather getting colder, the subjects in the control group engaged in less physical exercise in their daily lives, which led to a decline in self-control ability. Whatever, the causes of the decline of pain tolerance in the control group, our findings overall supports the positive effect that physical exercise can have on self-control ability.

The underlying mechanism of the positive effect of physical exercise may be related with specific changes in the brain. For example, it has been shown that increased aerobic fitness in childhood is associated with greater dorsal striatum volume, a region that facilitates cognitive control ability (Chaddock et al., 2010). However, research directly testing the mechanism underlying these effects could help answer questions of how long, how frequently, and what kinds of aerobic exercise should be performed in order to achieve optimal effects from physical exercise.

### Pain tolerance and modulation of attention

Previous studies have consistently shown that distractors are often good methods for improving pain tolerance in both clinical pain and lab-induced pain in both children and adults. Verhoeven et al. (2011) had 91 undergraduate students randomly assigned to (1) a distraction group, in which an attention-demanding tone-detection task was performed during the CPT, and (2) a control group, in which no distraction task was performed. Results showed that participants in the distraction group reported significantly less pain during the CPT. Swee and Schirmer (2015) provided evidence that even vocalization can help individuals cope with pain, and suggest that motoric processes more so than other processes, contribute to this effect. Jameson et al. (2011) suggested electronic gaming as a pain distraction method for children to improve pain tolerance, because an interactive distraction task (playing a game) includes greater central cognitive processing demands. Similarly, Wohlheiter and Dahlquist (2013) examined 3- to 6-years-old children who underwent three cold-pressor trials: one while receiving no intervention, one while playing a video game (interactive distraction), and one while watching a video game (passive distraction). Their findings suggest that young preschoolers can benefit from interactive distraction to manage acute pain.

In the present study, we found people had longer pain tolerance in CPT II than in CPT I, which may be caused by distraction effects. In CPT II, participants were instructed to complete the Stroop task while their hand was immersed in the cold water bath, having to modulate their attention between the two tasks. Thus, the Stroop task proved to be a good distractor for sensory pain, which may have led to the significant longer pain tolerance in CPT II than in CPT I.

We found a very similar effect of aerobic exercise on pain tolerance in both CPT I and CPT II (though relatively smaller effect size), suggesting that aerobic exercise could also improve self-control ability even in the less attention focused condition.

### Implications and shortages

Successful persistence in the CPT requires efforts and self-control. Pain tolerance can be facilitated by greater self-control abilities, especially in terms of emotional and behavioral self-control. Our results indicate that physical exercise can help train self-control to be less vulnerable to depletion in the pain condition.

The current study had some practical implications. Among various physical sports, running is a traditional form of aerobic exercise and has received widespread popularity across all ages, for it can be easily implemented and requires no special skills. Our results demonstrated that running may serve as a way to reduce people's vulnerability to depletion of self-control. Improved self-control stamina could help one to become more efficient in their daily life by improving the ability to deal with forthcoming affairs that call for self-control. Thus, future research studies are needed to explore physical exercise as a potential treatment for impulsive behavior stemming from compromised self-control, such as that seen in drug abuse, eating disorders, violence, and so on.

Moreover, for populations suffering from chronic pain, aerobic exercise may serve as an approach to help build both physical strength and self-control ability at the same time, helping to improve pain tolerance in daily life (Eccleston et al., 2014).

Despite interesting results, there is much work needed due to various limiting factors. First, the present study suffered from a small sample size. Future studies with larger samples are needed to verify our findings. Second, the participant pool in the current study contained only Chinese female college students. Similar studies with other populations are important in order to better generalize the findings. Third, we were unable to match intervention times for the two groups due to factors such as inclement weather, and participants from the control group completed the assigned exercises more frequently than those in the experimental group. Thus, we could perhaps postulate that the improvement in stamina found in the experimental group may be magnified if the two groups exercised with similar frequency in a future study. Last, we did not directly investigate the neural mechanisms underlying the effect of physical exercise on emotional and behavioral self-control after ego-depletion. Future research using functional magnetic resonance imaging (fMRI) and/or electroencephalography (EEG) techniques are necessary in order to address this need.

## Author contributions

ZZ, Substantial contributions to the conception or design of the work, interpretation of data for the work, revising it critically, Final approval of the version to be published. YL, acquisition, data analysis, interpretation of data, drafting the work. JX, preparation of materials, acquisition, analysis of data, interpretation of data. XH, contributions to the conception or design of the work, contributions to the interpretation, revising, Final approval of the version to be published.

### Conflict of interest statement

The authors declare that the research was conducted in the absence of any commercial or financial relationships that could be construed as a potential conflict of interest.
